# Anxiety disorders in patients with thyroid nodules vs. thyroid cancer: a retrospective cohort study

**DOI:** 10.3389/fendo.2025.1539442

**Published:** 2025-04-01

**Authors:** Edy Kornelius, Shih-Chang Lo, Chien-Ning Huang, Yu-Hsun Wang, Yi-Sun Yang

**Affiliations:** ^1^ School of Medicine, Chung Shan Medical University, Taichung, Taiwan; ^2^ Department of Internal Medicine, Division of Endocrinology and Metabolism, Chung Shan Medical University Hospital, Taichung, Taiwan; ^3^ Institute of Medicine, Chung Shan Medical University, Taichung, Taiwan; ^4^ Department of Medical Research, Chung Shan Medical University Hospital, Taichung, Taiwan

**Keywords:** thyroid nodule, thyroid cancer, anxiety, depression, mood disorder

## Abstract

**Background:**

Thyroid nodules, often discovered incidentally, typically require long-term monitoring and may contribute to psychological distress. Despite their prevalence, the psychological impact of thyroid nodules remains underexplored.

**Methods:**

This retrospective cohort study used data from the TrinetX platform (2010–2023), encompassing 118 million patients. Patients diagnosed with thyroid nodules were matched to those with thyroid cancer using propensity score matching for age, sex, race, socioeconomic status and comorbidities. The primary outcome was anxiety disorder risk, with secondary outcomes including depression, mood disorder, and insomnia.

**Results:**

After matching, 138,803 pairs were analyzed, with a mean age of 52 years, 70% female, and 66% White. Comorbidities were well-balanced. Patients with thyroid nodules had a significantly higher risk of developing anxiety disorder compared to those with thyroid cancer (HR 1.06; 95% CI: 1.03–1.08). Conversely, thyroid nodule patients had lower risks of depression (HR 0.93; 95% CI: 0.90–0.96), mood disorders (HR 0.95; 95% CI: 0.92–0.98), and insomnia (HR 0.93; 95% CI: 0.89–0.97). Psychotic disorders showed no significant difference (HR 1.03; 95% CI: 0.90–1.17).

**Conclusions:**

This study identifies a significant association between thyroid nodules and increased anxiety risk, while risks for depression, mood disorders, and insomnia were lower compared to thyroid cancer patients. Furthermore, a sensitivity analysis compared thyroid nodule patients to the general population and revealed elevated anxiety risk in patients with nodules, reinforcing that this increased risk is not solely attributable to cancer-related factors. Further research is warranted to confirm these findings and explore mechanisms underlying the psychological impact of thyroid nodules.

## 1 Introduction

Thyroid nodules are a common endocrine condition, with reported prevalence ranging from approximately 10% to 46% in the general population (1, 2). While many nodules are benign, their detection often necessitates long-term surveillance to monitor for potential malignant transformation. There is growing recognition that the need for ongoing monitoring can impose a significant psychological burden on patients (3). In fact, recent studies have demonstrated that patients with thyroid nodules experience greater anxiety and depression compared to individuals without nodules​ (4), suggesting that the very presence of a thyroid nodule can adversely impact mental health. Patients may face anxiety about the possibility of malignancy, repeated diagnostic procedures, and uncertainty about their health, all of which can contribute to stress.

Moreover, in the context of small thyroid cancers, studies comparing active surveillance with immediate surgery have yielded insights into patient well-being. One long-term study reported that patients who opted for surveillance maintained better overall quality of life and did not show increased anxiety over five years of follow-up compared to those who underwent early surgery (5). However, other research has suggested that living with an untreated thyroid lesion can still provoke considerable worry; for example, patients on active surveillance may experience fear of cancer progression and even decision regret regarding their management choice​ (6). These findings underscore that the psychological impact of long-term thyroid nodule monitoring is complex and not yet fully understood. In light of the limited and contradictory literature on this topic, further investigation is necessary to clarify the extent and drivers of psychological burden in this patient population.

Accordingly, the present study was undertaken to rigorously evaluate the mental health outcomes of patients undergoing prolonged thyroid nodule surveillance. We specifically sought to address two key research questions: (1) Does prolonged observation of thyroid nodules lead to significant anxiety or other forms of psychological distress in patients? and (2) What patient or nodule-related factors are associated with any such psychological distress during long-term monitoring? We hypothesized that patients under long-term surveillance would exhibit elevated anxiety levels, and that certain characteristics would correlate with greater psychological distress.

## 2 Methods

### 2.1 Study population

This retrospective cohort study utilized the TriNetX database, which comprises data from approximately 118 million patients, spanning from January 1, 2010, to December 31, 2023. TriNetX is a federated, cloud-based network that provides access to electronic medical records from a wide range of healthcare entities, including hospitals, primary care, and specialty treatment providers. The database includes diverse geographic locations, age groups, disease diagnoses, and medications. It aggregates patient data and offers statistical analysis results through a browser-based interface. With a waiver from the Western Institutional Review Board, TriNetX ensures that only aggregated counts and statistical summaries of de-identified data are used, avoiding access to protected health information and primary data collection in retrospective analyses. Additionally, this study received an exemption waiver from the Institutional Review Board (IRB) of Chung Shan Medical University Hospital, identified by the reference number CS2-20121.

To ensure comprehensive follow-up, we required at least two follow-up entries in the database. Eligible cases were those with a recorded diagnosis of thyroid nodules, with the cohort entry (index date) defined as the date of the first recorded diagnosis. Controls were identified as patients with a first-time diagnosis of thyroid cancer, with the index date similarly defined as the first available diagnosis date. Patients with a prior diagnosis of hyperthyroidism or hypothyroidism were excluded from the case. If a patient had both a thyroid nodule and thyroid cancer, they were classified under the control group (thyroid cancer group).

For the control group, patients with other thyroid diseases were not excluded. The severity of thyroid cancer is expected to be the primary driver of psychological outcomes, with the psychological impact of the cancer diagnosis likely dominating that of any coexisting thyroid disorder. Additionally, it is common for patients with thyroid cancer, particularly those undergoing thyroidectomy, to experience subsequent hypo- or hyperthyroidism due to necessary levothyroxine adjustments. This post-thyroidectomy adjustment period often results in temporary or fluctuating thyroid function levels as clinicians optimize dosing. Excluding these cases would reduce the generalizability of our findings, as post-surgical thyroid dysfunction is a frequent aspect of the thyroid cancer care pathway. Including these patients ensures a more representative sample, reflecting the complex, real-world presentation of thyroid cancer patients.

### 2.2 Selection strategy

The rationale for excluding patients with hyperthyroidism and hypothyroidism from the thyroid nodule group is based on the well-documented association of these conditions with a higher risk of depression and anxiety (7–12). This approach ensures that psychiatric outcomes are more specifically attributable to the presence of thyroid nodules rather than confounded by other thyroid dysfunctions that independently affect mental health.

We selected thyroid cancer as the comparison group, given that it is the malignant counterpart of benign thyroid nodules and is typically diagnosed with a high degree of accuracy. From a clinical perspective, the risk of psychiatric outcomes associated with benign thyroid nodules is unlikely to match that of thyroid cancer, providing a clear and meaningful distinction for comparative analysis.

Furthermore, to ensure that observed psychiatric outcomes are attributable to exposure in the case and control groups, we excluded patients with any prior psychiatric diagnosis before the index date. This step allows for a more precise evaluation of new psychiatric diagnoses following the identification of thyroid nodules or thyroid cancer.

### 2.3 Study outcomes

The primary outcome of this study was the first occurrence of an anxiety disorder, measured starting 90 days after the index date for both the thyroid nodule and thyroid cancer groups, and assessed using hazard ratios (HR). We used the 90-day cutoff to mitigate potential misclassification bias or acute psychological impact immediately following a cancer diagnosis.

Anxiety was chosen as the primary outcome due to its strong alignment with the uncertainty and prolonged surveillance associated with benign thyroid nodules. This ‘wait and watch’ approach often leads to heightened alertness, restlessness, and worry, as patients anticipate potential progression or malignancy despite a benign diagnosis. In addition to anxiety, the study also evaluated the risks of depression, mood disorder and insomnia as secondary outcomes. We selected psychotic disorders as a negative control based on their relatively distinct etiology and lower susceptibility to fluctuations in thyroid-related concerns, thereby helping assess whether the associations observed for anxiety and other disorders were specific to those conditions.

### 2.4 Disease coding and variables of interest

We identified diagnoses of thyroid nodules based on the presence of International Classification of Diseases, 10th Revision (ICD-10) codes E04.1 and E04.2, recorded on or after January 1, 2010. Thyroid cancer diagnoses were identified using ICD-10 code C73. Anxiety disorders were defined using ICD-10 codes F40–F48, while major depressive disorder was defined using ICD-10 code F32, mood disorders using ICD-10 codes F30–F39, and insomnia using ICD-10 code G47.

In addition to these diagnoses, several baseline characteristics and comorbidities were considered, including age, sex, race, socioeconomic status, diabetes, ischemic heart disease, and other chronic pain conditions. Further details on the specific criteria and classification of these comorbidities are provided in **Supplementary Table S1**.

To address potential data heterogeneity and ensure diagnostic accuracy, we utilized the TriNetX platform, which harmonizes data from diverse healthcare organizations by mapping to standardized terminologies such as ICD-10, Current Procedural Terminology (CPT), and Logical Observation Identifier Name and Codes (LOINC). This process facilitates consistency across the network. Diagnostic codes within TriNetX are derived from electronic health records and are assigned by licensed healthcare providers during patient encounters, reflecting professional clinical judgment. To mitigate misclassification bias, we included only patients with at least two separate diagnostic entries for each condition, thereby reducing the likelihood of errors due to transient or incorrect diagnoses. While these measures enhance data reliability, we acknowledge that inherent limitations in coding practices and data entry may still introduce some degree of bias.

### 2.5 Statistical analysis

We performed 1:1 propensity score matching (PSM) using the TriNetX platform’s default greedy nearest-neighbor method, without exact matching on specific covariates (13). Baseline characteristics were considered well matched if the standardized mean difference (SMD) was below 0.1 (14). For the analysis of anxiety disorder, PSM was applied to baseline characteristics such as age, sex, race, socio-economic status and comorbidities to ensure comparable cohort pairs. Cohort comparisons were conducted using the log-rank test, and the HR was calculated using a proportional hazards model. The null hypothesis was tested using a chi-square test to assess whether the cohorts were statistically equivalent. All statistical analyses were performed within the TriNetX platform, and statistical significance was defined as a two-sided p-value < 0.05.

Additionally, a subgroup analysis was conducted to assess the risk of anxiety disorders across different follow-up periods, including 1, 2, 3, 5, and 10 years. We also evaluate potential effect modification on the risk of anxiety associated with thyroid nodules, stratifying by age (<30, 30–60, and >60 years), sex (female vs. male), and race (White person, Black person, or Asian). Additionally, we assessed whether the presence of specific comorbidities was associated with an increased risk of anxiety.

Furthermore, we performed a sensitivity analysis comparing thyroid nodule patients with the general population to gain additional insight into the broader psychiatric impact of thyroid nodules. The flow diagram for this sensitivity analysis is presented in **Supplementary Figure S1**, with baseline characteristics in **Supplementary Table S2** and risk estimates in **Supplementary Table S3**.

## 3 Results

**Figure 1:**
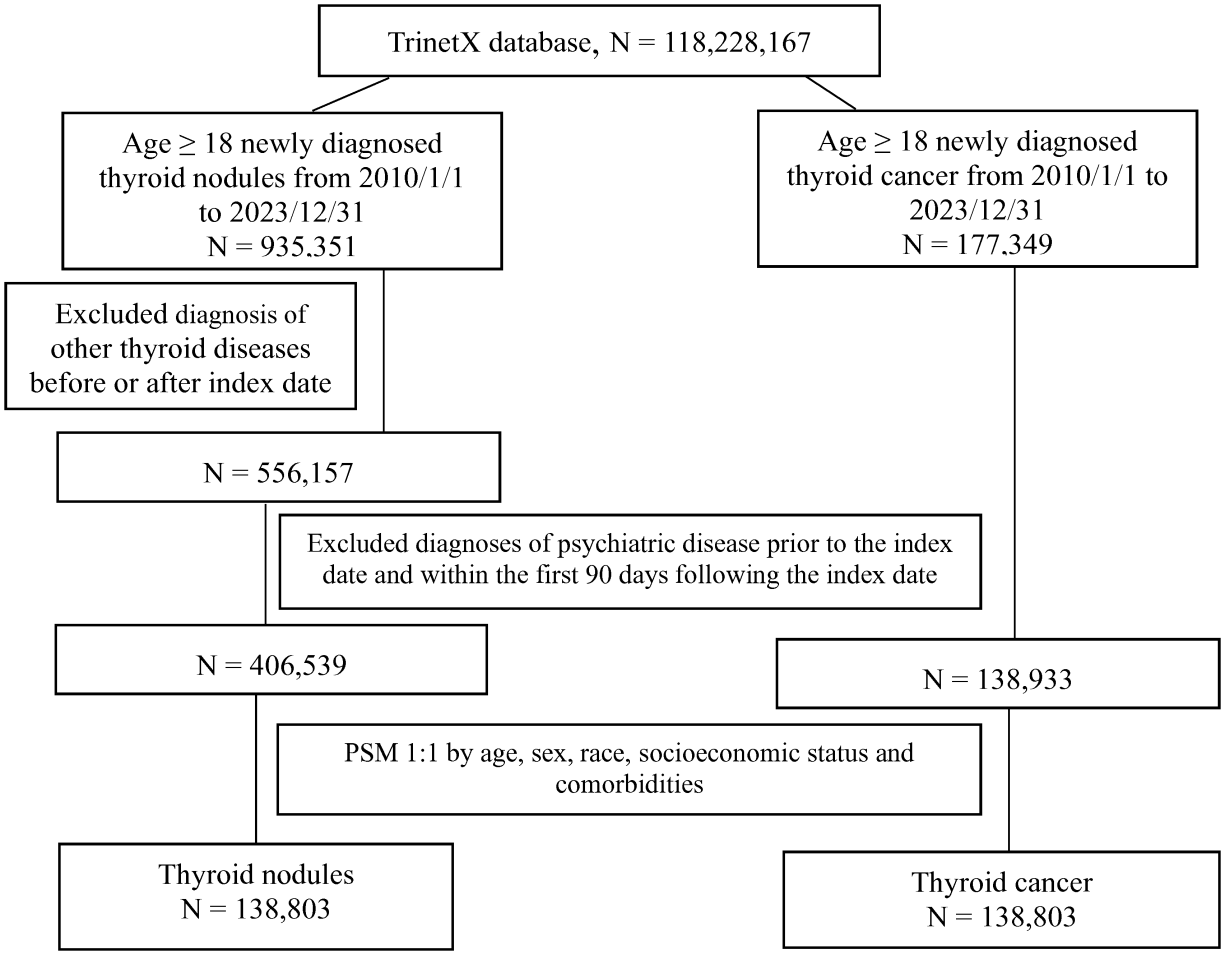
Flow chart of patient selection.

The complete flow diagram is shown in **Figure 1**. This study included a comprehensive cohort of 118 million patients. A total of 935,351 thyroid nodule and 177,349 thyroid cancer cases were initially identified. We excluded patients with hyperthyroidism and hypothyroidism, resulting in 556,157 thyroid nodule cases. Further exclusions were made for patients with a prior history of any psychiatric disease before the index date, leaving 406,539 thyroid nodule cases and 138,933 thyroid cancer controls. Finally, we applied PSM based on age, sex, race, socio-economic status and comorbidities, yielding 138,803 matched pairs for the final analysis.

**Table 1** presents the baseline demographic data for this study. Before PSM, baseline characteristics differed markedly between the thyroid nodule and thyroid cancer groups. The average age was 56.5 years for thyroid nodule patients and 51.9 years for thyroid cancer patients. Additionally, White constituted 59.1% of the thyroid nodule group and 66.5% of the thyroid cancer group, with various comorbidities also differing significantly between groups. After PSM, these differences were well-balanced, confirmed by a standardized mean difference (SMD) of less than 0.1. The matched cohort had a mean age of 52 years, 70% female representation, and 66% White. Socioeconomic status and comorbidities were also balanced, with rates of overweight and obesity at 4%, type 2 diabetes at 5%, heart disease at 4.5%, and neoplasms at 14.3%.

**Table 1:** Baseline characteristics of the thyroid nodule and thyroid cancer cases.

	Before PSM		Std diff.	After PSM		Std diff.
	Thyroid nodules N = 406539	Thyroid cancer N = 138933		Thyroid nodules N = 138803	Thyroid cancer N = 138803	
Age, years	56.55 ± 15.72	51.95 ± 15.45	**0.295**	52.00 ± 15.58	51.96 ± 15.45	0.002
Sex						
Female	289025 (71.09)	97800 (70.39)	0.015	97485 (70.23)	97705 (70.39)	0.003
Male	103589 (25.48)	36854 (26.53)	0.024	37081 (26.72)	36819 (26.53)	0.004
Race						
White	240269 (59.10)	92337 (66.46)	**0.153**	92492 (66.64)	92309 (66.50)	0.003
Black or African American	55475 (13.65)	8995 (6.47)	**0.240**	9056 (6.52)	8995 (6.48)	0.002
Asian	21796 (5.36)	7807 (5.62)	0.011	7764 (5.59)	7807 (5.63)	0.001
Native Hawaiian or Other Pacific Islander	2466 (0.61)	960 (0.69)	0.011	883 (0.64)	959 (0.69)	0.007
American Indian or Alaska Native	1288 (0.32)	1156 (0.83)	0.068	1000 (0.72)	1056 (0.76)	0.005
Unknown Race	68036 (16.74)	21178 (15.24)	0.041	21058 (15.17)	21178 (15.26)	0.002
Marital status						
Married	108787 (26.76)	37377 (26.90)	0.003	37579 (27.07)	37367 (26.92)	0.003
Never Married	39867 (9.81)	11455 (8.25)	0.055	11290 (8.13)	11452 (8.25)	0.004
Divorced	15515 (3.82)	4448 (3.20)	0.033	4442 (3.20)	4447 (3.20)	0.000
Widowed	17668 (4.35)	4325 (3.11)	0.065	4274 (3.08)	4324 (3.12)	0.002
Legally Separated	1764 (0.43)	504 (0.36)	0.011	462 (0.33)	504 (0.36)	0.005
Domestic partner	954 (0.24)	363 (0.26)	0.005	333 (0.24)	362 (0.26)	0.004
Persons with potential health hazards related to socioeconomic and psychosocial circumstances	1020 (0.25)	206 (0.15)	0.023	168 (0.12)	206 (0.15)	0.007
Problems related to education and literacy	64 (0.02)	17 (0.01)	0.003	10 (0.01)	17 (0.01)	0.005
Problems related to employment and unemployment	74 (0.02)	19 (0.01)	0.004	10 (0.01)	19 (0.01)	0.006
Problems related to housing and economic circumstances	334 (0.08)	47 (0.03)	0.020	47 (0.03)	47 (0.03)	0.000
Problems related to social environment	173 (0.04)	30 (0.02)	0.012	32 (0.02)	30 (0.02)	0.001
Problems related to upbringing	10 (0.00)	10 (0.01)	0.007	10 (0.01)	10 (0.01)	0.000
Other problems related to primary support group, including family circumstances	170 (0.04)	42 (0.03)	0.006	25 (0.02)	42 (0.03)	0.008
Problems related to certain psychosocial circumstances	37 (0.01)	15 (0.01)	0.002	11 (0.01)	15 (0.01)	0.003
Problems related to other psychosocial circumstances	148 (0.04)	33 (0.02)	0.007	24 (0.02)	33 (0.02)	0.005
Problems related to lifestyle	3042 (0.75)	570 (0.41)	0.045	410 (0.30)	570 (0.41)	0.019
Family history of mental and behavioral disorders	105 (0.03)	22 (0.02)	0.007	16 (0.01)	22 (0.02)	0.004
Personal history of psychological trauma, not elsewhere classified	12 (0.00)	10 (0.01)	0.006	10 (0.01)	10 (0.01)	0.000
Personal history of self-harm	10 (0.00)	10 (0.01)	0.007	10 (0.01)	10 (0.01)	0.000
Alcohol related disorders	1565 (0.39)	268 (0.19)	0.036	219 (0.16)	268 (0.19)	0.008
Nicotine dependence	10765 (2.65)	2031 (1.46)	0.084	1750 (1.26)	2031 (1.46)	0.017
Overweight and obesity	19272 (4.74)	5653 (4.07)	0.033	5166 (3.72)	5650 (4.07)	0.018
Cerebral infarction	4443 (1.09)	600 (0.43)	0.076	524 (0.38)	600 (0.43)	0.009
Behavioral syndromes associated with physiological disturbances and physical factors	952 (0.23)	224 (0.16)	0.016	157 (0.11)	224 (0.16)	0.013
Disorders of adult personality and behavior	87 (0.02)	23 (0.02)	0.004	18 (0.01)	23 (0.02)	0.003
Cannabis related disorders	499 (0.12)	94 (0.07)	0.018	79 (0.06)	94 (0.07)	0.004
Opioid related disorders	450 (0.11)	69 (0.05)	0.022	66 (0.05)	69 (0.05)	0.001
Cocaine related disorders	223 (0.06)	24 (0.02)	0.020	28 (0.02)	24 (0.02)	0.002
Other stimulant related disorders	131 (0.03)	24 (0.02)	0.010	22 (0.02)	24 (0.02)	0.001
Other psychoactive substance related disorders	373 (0.09)	52 (0.04)	0.021	62 (0.05)	52 (0.04)	0.004
Neoplasms	53187 (13.08)	21198 (15.26)	0.062	19868 (14.31)	21161 (15.25)	0.026
Type 2 diabetes mellitus	31241 (7.69)	7169 (5.16)	**0.103**	6863 (4.94)	7169 (5.17)	0.010
Other forms of heart disease	30902 (7.60)	6770 (4.87)	**0.113**	6321 (4.55)	6768 (4.88)	0.015
Chronic pain, not elsewhere classified	9976 (2.45)	1818 (1.31)	0.084	1655 (1.19)	1818 (1.31)	0.011

Std diff., standardized mean difference; PSM, propensity score matching. Race and ethnicity classifications were derived from clinical records as documented by medical professionals in the TriNetX database. Bold values indicate statistically significant.

**Table 2** presents the primary outcomes of this study. Patients with thyroid nodules demonstrated a consistently higher risk of developing anxiety compared to those with thyroid cancer, with an overall HR of 1.06 (95% CI: 1.03–1.08). Conversely, patients with thyroid nodules had a lower risk of depression (HR 0.93; 95% CI: 0.90–0.96), mood disorders (HR 0.95; 95% CI: 0.92–0.98), and insomnia (HR 0.93; 95% CI: 0.89–0.97) than those with thyroid cancer, with similar trends persisting across follow-up intervals (**Table 3**). Psychotic disorders showed no significant difference between groups (HR 1.03; 95% CI: 0.90–1.17).

**Table 2:** Risk of all psychiatric illness exposed to thyroid nodules compared with thyroid cancer (n = 138,308 matched pairs).

	HR (95% C.I.)
Anxiety	1.06 (1.03–1.08)
Depression	0.93 (0.90–0.96)
Mood disorders	0.95 (0.92–0.98)
Insomnia	0.93 (0.89–0.97)
Psychotic disorders	1.03 (0.90–1.17)

**Table 3:** Subgroup Analysis of psychiatric disease in patients with thyroid nodules compared to thyroid cancer across different follow-up durations.

	1 year	2 years	3 years	5 years	10 years
Anxiety	1.04 (0.99–1.09)	1.07 (1.03–1.11)	1.08 (1.05–1.12)	1.07 (1.04–1.10)	1.06 (1.03–1.08)
Depression	0.85 (0.80–0.91)	0.90 (0.85–0.94)	0.91 (0.87–0.95)	0.92 (0.88–0.95)	0.93 (0.90–0.96)
Mood disorders	0.88 (0.83–0.94)	0.92 (0.88–0.96)	0.94 (0.90–0.97)	0.94 (0.91–0.98)	0.95 (0.93–0.98)
Insomnia	0.86 (0.78–0.95)	0.88 (0.83–0.95)	0.89 (0.84–0.95)	0.93 (0.88–0.98)	0.93 (0.89–0.98)
Psychotic disorders	0.92 (0.69–1.23)	1.00 (0.80–1.23)	1.02 (0.85–1.23)	1.00 (0.85–1.17)	1.04 (0.91–1.20)

Stratified analysis revealed that patients with thyroid nodules had varying psychiatric risks compared to those with thyroid cancer, influenced by age, sex, race, and comorbidities (**Table 4**). Younger patients (<30 years) with thyroid nodules exhibited notably higher risks of anxiety (HR 1.31; 95% CI: 1.21–1.41) and mood disorders (HR 1.15; 95% CI: 1.04–1.26) than their thyroid cancer counterparts, whereas patients over 60 years showed a reduced risk for anxiety (HR 0.97; 95% CI: 0.93–1.02) and depression (HR 0.89; 95% CI: 0.84–0.94). Females with thyroid nodules demonstrated a higher risk of anxiety (HR 1.07; 95% CI: 1.04–1.10) compared to those with thyroid cancer, while male patients showed no significant differences across psychiatric outcomes. Racial stratification indicated that White patients with thyroid nodules had an increased risk of anxiety (HR 1.09; 95% CI: 1.06–1.12), whereas Black/African American patients exhibited lower risks for depression (HR 0.86; 95% CI: 0.77–0.97) and mood disorders (HR 0.85; 95% CI: 0.76–0.95) relative to the thyroid cancer group.

**Table 4:** Stratification for risk of psychiatric disease exposed to thyroid nodules compared to thyroid cancer.

	Thyroid nodules vs. Thyroid cancer-HR (95% C.I.)				
	Anxiety	Depression	Mood disorders	Insomnia	Psychotic disorders
Age					
<30	**1.31 (1.21–1.41)**	1.09 (0.98–1.21)	**1.15 (1.04–1.26)**	0.96 (0.79–1.16)	0.89 (0.52–1.50)
30-60	**1.09 (1.06–1.12)**	**0.94 (0.90–0.98)**	**0.95 (0.91–0.98)**	0.97 (0.91–1.02)	1.16 (0.97–1.39)
>60	0.97 (0.93–1.02)	**0.89 (0.84–0.94)**	**0.91 (0.86–0.96)**	**0.90 (0.84–0.97)**	0.83 (0.67–1.05)
Sex					
Female	**1.07 (1.04–1.10)**	**0.93 (0.90–0.97)**	**0.95 (0.92–0.98)**	**0.94 (0.89–0.99)**	1.09 (0.92–1.30)
Male	1.05 (0.99–1.11)	1.00 (0.93–1.07)	1.00 (0.94–1.07)	0.94 (0.87–1.02)	0.91 (0.71–1.17)
Race					
White	**1.09 (1.06–1.12)**	**0.95 (0.91–0.98)**	0.97 (0.94–1.002)	0.98 (0.93–1.03)	1.13 (0.95–1.33)
Black/African American	0.96 (0.88–1.05)	**0.86 (0.77–0.97)**	**0.85 (0.76–0.95)**	0.92 (0.79–1.07)	0.86 (0.60–1.23)
Asian	1.05 (0.92–1.20)	1.05 (0.86–1.27)	1.06 (0.89–1.27)	0.97 (0.81–1.17)	0.95 (0.44–2.07)
Comorbidities					
Nicotine dependence	1.05 (0.90–1.23)	1.00 (0.83–1.21)	1.00 (0.83–1.19)	1.03 (0.78–1.36)	1.28 (0.63–2.64)
Overweight and obesity	0.98 (0.88–1.08)	1.00 (0.88–1.13)	0.96 (0.85–1.08)	**0.79 (0.67–0.94)**	0.67 (0.37–1.22)
Neoplasms	1.03 (0.98–1.09)	**0.90 (0.83–0.97)**	**0.90 (0.84–0.96)**	0.95 (0.86–1.03)	1.09 (0.81–1.46)
Type 2 diabetes mellitus	1.03 (0.94–1.13)	0.96 (0.86–1.06)	0.94 (0.85–1.04)	1.07 (0.93–1.24)	0.80 (0.54–1.17)
Other forms of heart	0.97 (0.87–1.07)	**0.83 (0.73–0.95)**	**0.83 (0.73–0.93)**	0.93 (0.79–1.08)	0.79 (0.50–1.23)
disease					

Race and ethnicity classifications were derived from clinical records as documented by medical professionals in the TriNetX database. Bold values indicate statistically significant.

In the additional sensitivity analysis comparing thyroid nodule patients (n=335,954) to a matched general population without thyroid disease, we observed a significantly higher risk of both anxiety (HR 1.36; 95% CI: 1.34–1.38) and mood disorders (HR 1.16; 95% CI: 1.14–1.18). This finding supports the notion that the increased anxiety risk among thyroid nodule patients exists independently of cancer-specific factors, underscoring the unique psychological burden of nodule surveillance. Detailed data are presented in **Supplementary Tables S2**, **S3**.

## 4 Discussion

To the best of our knowledge, this is the largest retrospective study to evaluate the risk of anxiety disorders in patients with thyroid nodules. Despite using thyroid cancer as an extreme control group, our findings reveal that patients with thyroid nodules exhibit a significantly higher risk of developing anxiety disorders compared to those with thyroid cancer, with an overall HR for anxiety of 1.06 (95% CI: 1.03–1.08). This risk remained consistent across various stratifications, including age, sex, and race. Importantly, the study’s reliability is further supported by the lack of an increased risk for psychotic disorders, included as a negative control, which strengthens the validity of our anxiety findings.

Additionally, the year-by-year subgroup analyses confirmed the stability of these results over time, indicating that the association between thyroid nodules and anxiety persists in long-term follow-up. In contrast, patients with thyroid nodules demonstrated a lower risk for other psychiatric outcomes, including depression, mood disorders, and insomnia, relative to thyroid cancer patients. One potential explanation is that a thyroid cancer diagnosis may prompt more frequent healthcare visits and screening for psychiatric comorbidities, thereby increasing the detection of conditions such as depression or insomnia. Alternatively, post-surgical or cancer-related distress could manifest in different psychiatric domains, contributing to higher rates of depression in the thyroid cancer cohort (6).

When compared to the general population in our sensitivity analysis, patients with thyroid nodules had elevated risks of both anxiety (HR 1.36; 95% CI: 1.34–1.38) and mood disorder (HR 1.16; 95% CI: 1.14–1.18). These findings suggest that while thyroid nodules confer a heightened overall risk for psychiatric conditions, the contrasting results observed when comparing nodules to thyroid cancer reflect distinct psychological contexts across different reference groups.

The literature on the relationship between thyroid nodules and psychiatric outcomes is limited, with most studies focusing on thyroid cancer rather than benign thyroid conditions. In a study conducted in China, Li et al. examined distress and sleep disturbance among adults undergoing thyroid nodule screening, diagnosis, and treatment, finding increased psychological distress and sleep issues among those with thyroid nodules (3). Interestingly, Li et al. also explored psychiatric outcomes in thyroid cancer patients, noting a worsening in psychological outcomes. However, our findings contrast with theirs, as we observed that thyroid cancer patients did not exhibit a higher risk of psychiatric illness compared to those with benign thyroid nodules. One potential explanation for this discrepancy may lie in the relatively short follow-up period in the Li et al. study, which might capture only initial psychiatric impacts; in contrast, the extended surveillance often required for thyroid nodules may lead to a sustained, gradual increase in psychiatric risk over time.

Most research on psychological and quality of life impacts in thyroid disorders has centered on thyroid cancer (15–18). For example, studies from Korea assessed quality of life in patients with papillary thyroid microcarcinoma (PTMC), comparing outcomes between active surveillance and surgical intervention (19, 20). Jeon et al. reported that PTMC patients under active surveillance had fewer health-related problems than those who underwent surgery, supporting active surveillance as a viable management option (19). Similarly, Kong et al. found that patients opting for surveillance experienced better psychological and physical health both at baseline and during follow-up, compared to those who underwent immediate surgery (20). While these studies provide valuable insights into managing thyroid cancer, they do not address the unique psychological challenges faced by patients with benign thyroid nodules.

The exact mechanisms underlying the elevated risk of anxiety observed in patients with benign thyroid nodules remain unknown. However, one primary contributing factor may be the inherent uncertainty and prolonged surveillance associated with benign thyroid nodules, often requiring years of regular imaging and follow-up. This ongoing monitoring can lead to anticipatory anxiety, as patients frequently worry about potential nodule progression or malignancy despite the benign diagnosis. In contrast to thyroid cancer, where treatment protocols are typically well-defined, the ‘wait and watch’ approach often recommended for thyroid nodules may impose a sustained psychological burden, heightening patient vigilance and alertness to somatic changes.

Durante et al. demonstrated that among patients with asymptomatic, sonographically or cytologically benign thyroid nodules, most nodules showed no significant size increase over a 5-year follow-up period, and cases of thyroid cancer were rare (21). Current guidelines recommend initial ultrasound follow-up intervals of 6 to 18 months, with less frequent monitoring if the nodule remains stable (22). Additionally, recent updates suggest re-evaluation at 3 to 5 years, with the potential to reclassify or classify nodules as stable, allowing for discontinuation of further follow-up in many cases (23). This structured, extended monitoring can contribute to anticipatory anxiety, emphasizing the need for psychological support alongside routine clinical follow-up.

This study has several notable strengths. It represents the largest retrospective analysis to date on psychiatric risks in patients with benign thyroid nodules, leveraging a large cohort to enhance generalizability. Furthermore, the extended follow-up period, including year-by-year analyses, allowed us to observe trends in psychiatric risk over time, lending greater reliability to our findings. We also utilized a negative control outcome to strengthen causal inference regarding the specificity of the findings.

Nevertheless, the use of EHR data from multiple institutions introduces heterogeneity and potential coding inconsistencies, which may lead to misclassification of both thyroid conditions and psychiatric outcomes. Additionally, although PSM balanced many observable variables, unmeasured confounders (e.g., baseline psychological state, social support systems, and lifestyle factors) could still influence the results.

The findings of this study suggest several important areas for further investigation. First, more rigorous prospective studies or randomized controlled trials are needed to confirm the observed association between thyroid nodules and anxiety, ideally using standardized, validated psychiatric assessment tools. Future research should also investigate thyroid nodule characteristics—such as size, location, duration, or biopsy frequency—to evaluate whether certain subgroups have a heightened psychological risk. Finally, mechanistic studies examining potential hormonal, immunological, or psychosocial pathways are warranted to elucidate how benign thyroid nodules might precipitate or exacerbate psychiatric disorders.

In conclusion, this study demonstrates a significant association between benign thyroid nodules and an elevated risk of anxiety disorder, highlighting the importance of recognizing and addressing psychological distress in these patients. While thyroid nodule patients showed higher anxiety than even those with thyroid cancer, they paradoxically exhibited lower risks of depression, mood disorders, and insomnia relative to the cancer group. Compared to the general population, however, patients with thyroid nodules also displayed increased risks for both anxiety and mood disorders, underscoring the independent psychological burden posed by benign nodule surveillance. These findings underscore the need for clinicians to provide comprehensive education, reassurance regarding the typically benign nature of nodules, and, where appropriate, referrals to mental health professionals or the use of stress management techniques. Further prospective validation is essential to refine these recommendations and tailor interventions effectively.

## Data Availability

This population-based study obtained data from the TrinetX platform (accessible at https://trinetx.com/), for which third-party restrictions apply to the availability of this data. The data were used under license for this study with restrictions that do not allow for data to be redistributed or made publicly available. To gain access to the data, a request can be made to TriNetX (join@trinetx.com), but costs might be incurred, and a data-sharing agreement would be necessary.

## Supplementary material

Supplementary Figure 1 Sensitivity Analysis Comparing Patients with Thyroid Nodules to the General Population.
