# Supplementary figures and images for: Anxiety disorders in patients with thyroid nodules vs. thyroid cancer: a retrospective cohort study
Source: Front Endocrinol (Lausanne). 2025 Apr 1;16:1539442. doi: 10.3389/fendo.2025.1539442 (PMC11996671; doi:10.3389/fendo.2025.1539442)

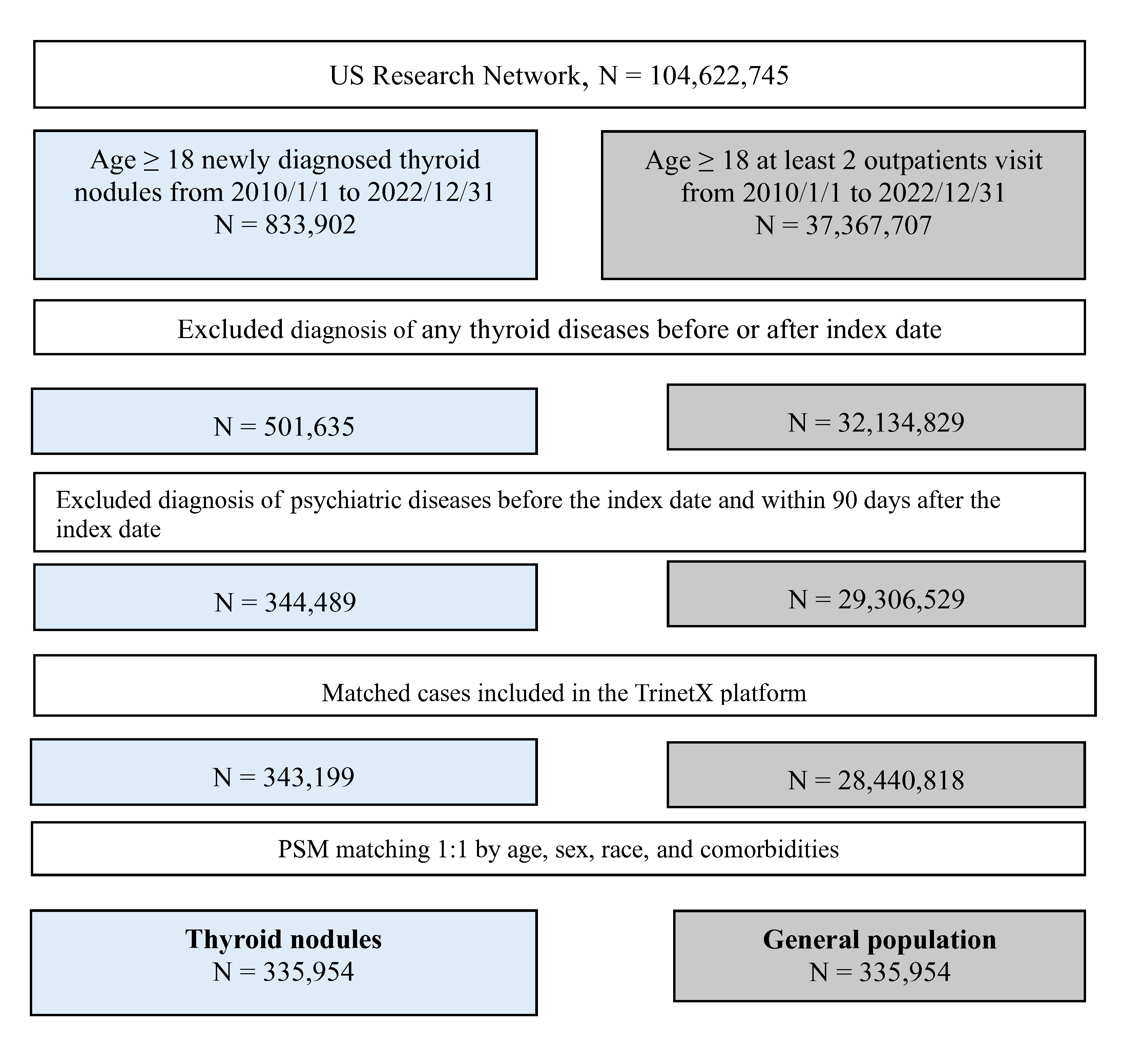

Supplement: Supplementary Figure 1 — Sensitivity Analysis Comparing Patients with Thyroid Nodules to the General Population. [file Image1.tif]
