# Supplementary material for: Anxiety disorders in patients with thyroid nodules vs. thyroid cancer: a retrospective cohort study
Source: Front Endocrinol (Lausanne). 2025 Apr 1;16:1539442. doi: 10.3389/fendo.2025.1539442 (PMC11996671; doi:10.3389/fendo.2025.1539442)
Supplement: Supplementary file 2 [file DataSheet1.docx]

| **Supplemental table 1**. Detailed coding of this study | |
| --- | --- |
| **Study outcome** |  |
| Anxiety | Anxiety, dissociative, stress-related, somatoform and other nonpsychotic mental disorder (ICD-10: F40-F48) |
| Depression | Depressive episode (ICD-10 code: F32) |
| Mood disorders | Mood [affective] disorders (ICD-10 code: F30-F39) |
| Insomnia | Insomnia (ICD-10 code: G47.0) |
| Psychotic disorders | Schizophrenia, schizotypal, delusional, and other non-mood psychotic disorders (ICD-10 : F20-F29) |
|  |  |
| **Cases** |  |
| Thyroid nodules | Nontoxic single thyroid nodule (ICD-10: E04.1) |
|  | Nontoxic multinodular goiter (ICD-10: E04.2) |
|  |  |
| **Controls** |  |
| Thyroid cancer | Malignant neoplasms of thyroid cancer (ICD-10: C73) |
|  |  |
| **Exclusions:** |  |
| Thyroid related diseases | Malignant neoplasms of thyroid and other endocrine glands (ICD10CM:C73-C75) |
|  | Personal history of malignant neoplasm of endocrine glands (ICD10CM:Z85.85) |
|  | Thyrotoxicosis [hyperthyroidism] (ICD10CM:E05) |
|  | Endocrine, nutritional and metabolic diseases complicating pregnancy, unspecified trimester (ICD10CM:O99.280) |
|  | Chronic thyroiditis with transient thyrotoxicosis (ICD10CM:E06.2) |
|  | Thyroiditis (ICD10CM:E06) |
|  | Other hypothyroidism (ICD10CM:E03) |
|  | Endocrine, nutritional and metabolic diseases complicating pregnancy, first trimester (ICD10CM:O99.281) |
|  | Postprocedural hypothyroidism (ICD10CM:E89.0) |
|  | Endocrine, nutritional and metabolic diseases complicating childbirth (ICD10CM:O99.284) |
|  | Other iodine-deficiency related thyroid disorders and allied conditions (ICD10CM:E01.8) |
|  | Endocrine, nutritional and metabolic diseases complicating the puerperium (ICD10CM:O99.285) |
|  | Congenital iodine-deficiency syndrome (ICD10CM:E00) |
|  | Neuropathic heredofamilial amyloidosis (ICD10CM:E85.1) |
|  | Subclinical iodine-deficiency hypothyroidism (ICD10CM:E02) |
|  | Iodine-deficiency related thyroid disorders and allied conditions (ICD10CM:E01) |
|  | Other specified disorders involving the immune mechanism, not elsewhere classified (ICD10CM:D89.89) |
|  |  |
| **Comorbidities** |  |
| Socioeconomic problems | Persons with potential health hazards related to socioeconomic and psychosocial circumstances (ICD10CM: Z55-Z65) |
|  | Problems related to education and literacy (ICD10CM:Z55) |
|  | Problems related to employment and unemployment (ICD10CM:Z56) |
|  | Problems related to housing and economic circumstances (ICD10CM:Z59) |
|  | Problems related to social environment (ICD10CM:Z60) |
|  | Problems related to upbringing (ICD10CM:Z62) |
|  | Other problems related to primary support group, including family circumstances ((ICD10CM:Z63) |
|  | Problems related to certain psychosocial circumstances (ICD10CM:Z64) |
|  | Problems related to other psychosocial circumstances (ICD10CM:Z65) |
|  | Problems related to lifestyle (ICD10CM:Z72) |
|  | Family history of mental and behavioral disorders (ICD10CM:Z81) |
|  | Personal history of psychological trauma, not elsewhere classified (ICD10CM:Z91.4) |
|  | Personal history of self-harm (ICD10CM: Z91.5) |
| Alcohol | Alcohol related disorders (ICD10CM:F10) |
| Smoking | Nicotine dependence (ICD10CM:F17) |
| Overweight and obesity | Overweight and obesity (ICD10CM:E66) |
| Cerebral infarction | Cerebral infarction (ICD10CM: I63) |
| Behaviour syndromes | Behavioral syndromes associated with physiological disturbances and physical factors (ICD10CM:F50-F59) |
|  | Disorders of adult personality and behavior (ICD10CM:F60-F69) |
| Substance abuse | Cannabis related disorders (ICD10CM:F12) |
|  | Opioid related disorders (ICD10CM:F11) |
|  | Cocaine related disorders (ICD10CM:F14) |
|  | Other stimulant related disorders (ICD10CM:F15) |
|  | Other psychoactive substance related disorders (ICD10CM:F19) |
| Neoplasm | Neoplasms (ICD10CM:C00-D49) |
| Type 2 diabetes | Type 2 diabetes mellitus (ICD10CM:E11) |
| Other forms of heart diseases | Other forms of heart disease (ICD10CM:I30-I5A) |
| Chronic pain | Chronic pain, not elsewhere classified (ICD10CM:G89.2) |

**Supplemental Table 2.** Baseline characteristics of the thyroid nodule and general population

|  | Before PSM | |  |  |  | After PSM | |  |  |
| --- | --- | --- | --- | --- | --- | --- | --- | --- | --- |
|  | Thyroid nodules | General population | p | Std diff. |  | Thyroid nodules | General population | p | Std diff. |
| N | 335954 | 28440818 |  |  |  | 335954 | 335954 |  |  |
| Age, years | 56.7 ± 15.7 | 47.1 ± 17.5 | <0.001 | 0.579 |  | 56.7 ± 15.7 | 56.7 ± 15.7 | 1 | <0.001 |
| Female | 243115 (72.4) | 54252 (0.2) | <0.001 | 0.404 |  | 243115 (72.4) | 241728 (72.0) | <0.001 | 0.009 |
| Race |  |  |  |  |  |  |  |  |  |
| White | 207030 (61.6) | 17750362 (62.4) | <0.001 | 0.016 |  | 207030 (61.6) | 211895 (63.1) | <0.001 | 0.030 |
| Black or African American | 45715 (13.6) | 3583506 (12.6) | <0.001 | 0.030 |  | 45715 (13.6) | 44383 (13.2) | <0.001 | 0.012 |
| Asian | 16395 (4.9) | 1068286 (3.8) | <0.001 | 0.055 |  | 16395 (4.9) | 12768 (3.8) | <0.001 | 0.053 |
| Native Hawaiian or other Pacific islander | 1466 (0.4) | 94431 (0.3) | <0.001 | 0.017 |  | 1466 (0.4) | 1241 (0.4) | <0.001 | 0.011 |
| American Indian or Alaska Native | 1023 (0.3) | 109669 (0.4) | <0.001 | 0.014 |  | 1023 (0.3) | 1121 (0.3) | 0.034 | 0.005 |
| Diabetes |  |  |  |  |  |  |  |  |  |
| Type 1 | 1774 (0.5) | 25713 (0.1) | <0.001 | 0.079 |  | 1774 (0.5) | 885 (0.3) | <0.001 | 0.042 |
| Type 2 | 26420 (7.9) | 328114 (1.2) | <0.001 | 0.328 |  | 26420 (7.9) | 18134 (5.4) | <0.001 | 0.099 |
| Overweight and obesity | 15804 (4.7) | 174877 (0.6) | <0.001 | 0.256 |  | 15804 (4.7) | 7329 (2.2) | <0.001 | 0.139 |
| Hypertensive disease | 58735 (17.5) | 732788 (2.6) | <0.001 | 0.512 |  | 58735 (17.5) | 58735 (17.5) | 1 | <0.001 |
| Other forms of heart disease | 26444 (7.9) | 352203 (1.2) | <0.001 | 0.322 |  | 26444 (7.9) | 17047 (5.1) | <0.001 | 0.114 |
| Nicotine dependence | 9633 (2.9) | 263138 (0.9) | <0.001 | 0.143 |  | 9633 (2.9) | 6483 (1.9) | <0.001 | 0.061 |
| Alcohol related disorders | 1643 (0.5) | 73858 (0.3) | <0.001 | 0.038 |  | 1643 (0.5) | 1572 (0.5) | 0.209 | 0.003 |
| Chronic kidney diseases |  |  |  |  |  |  |  |  |  |
| Chronic kidney disease | 10631 (3.2) | 101930 (0.4) | <0.001 | 0.215 |  | 10631 (3.2) | 6273 (1.9) | <0.001 | 0.083 |
| Hypertensive chronic kidney disease | 4471 (1.3) | 35617 (0.1) | <0.001 | 0.142 |  | 4471 (1.3) | 3138 (0.9) | <0.001 | 0.038 |
| Chronic liver disease |  |  |  |  |  |  |  |  |  |
| Alcoholic liver disease | 318 (0.1) | 12953 (0.0) | <0.001 | 0.019 |  | 318 (0.1) | 291 (0.1) | 0.274 | 0.003 |
| Hepatic failure, not elsewhere classified | 420 (0.1) | 9685 (0.0) | <0.001 | 0.032 |  | 420 (0.1) | 300 (0.1) | <0.001 | 0.011 |
| Chronic hepatitis, not elsewhere classified | 77 (0.0) | 1221 (0.0) | <0.001 | 0.016 |  | 77 (0.0) | 30 (0.0) | <0.001 | 0.011 |
| Fibrosis and cirrhosis of liver | 1465 (0.4) | 21974 (0.1) | <0.001 | 0.071 |  | 1465 (0.4) | 649 (0.2) | <0.001 | 0.043 |
| Cerebral infarction | 4184 (1.2) | 57784 (0.2) | <0.001 | 0.123 |  | 4184 (1.2) | 3438 (1.0) | <0.001 | 0.021 |
| Dementia |  |  |  |  |  |  |  |  |  |
| Vascular dementia | 176 (0.1) | 1452 (0.0) | <0.001 | 0.028 |  | 176 (0.1) | 155 (0.0) | 0.248 | 0.003 |
| Unspecified dementia | 834 (0.2) | 8877 (0.0) | <0.001 | 0.058 |  | 834 (0.2) | 872 (0.3) | 0.357 | 0.002 |
| Dementia in other diseases classified  elsewhere | 377 (0.1) | 3740 (0.0) | <0.001 | 0.040 |  | 377 (0.1) | 351 (0.1) | 0.335 | 0.002 |
| Alzheimer's disease | 330 (0.1) | 3600 (0.0) | <0.001 | 0.036 |  | 330 (0.1) | 313 (0.1) | 0.502 | 0.002 |
| Neoplasms |  |  |  |  |  |  |  |  |  |
| Neoplasms | 45471 (13.5) | 442280 (1.6) | <0.001 | 0.466 |  | 45471 (13.5) | 11685 (3.5) | <0.001 | 0.366 |
| Malignant neoplasms of lymphoid,  hematopoietic and related tissue | 3784 (1.1) | 34292 (0.1) | <0.001 | 0.128 |  | 3784 (1.1) | 918 (0.3) | <0.001 | 0.102 |
| Psoriasis | 1329 (0.4) | 16486 (0.1) | <0.001 | 0.071 |  | 1329 (0.4) | 446 (0.1) | <0.001 | 0.051 |
| Rheumatoid arthritis |  |  |  |  |  |  |  |  |  |
| Rheumatoid arthritis with rheumatoid factor | 705 (0.2) | 1768 (0.0) | <0.001 | 0.062 |  | 705 (0.2) | 47 (0.0) | <0.001 | 0.059 |
| Other rheumatoid arthritis | 2277 (0.7) | 22914 (0.1) | <0.001 | 0.097 |  | 2277 (0.7) | 1010 (0.3) | <0.001 | 0.054 |
| Systemic lupus erythematosus | 803 (0.2) | 8349 (0.0) | <0.001 | 0.057 |  | 803 (0.2) | 255 (0.1) | <0.001 | 0.041 |

Std diff.: standardized mean difference; PSM: propensity score matching. Race and ethnicity classifications were derived from clinical records as documented by medical professionals in the TriNetX database.

**Supplemental Table 3.** Sensitivity analysis evaluating the risk of anxiety in patients with thyroid nodules compared to the general population.

|  | No. of event | | Incidence (%) | |  | |
| --- | --- | --- | --- | --- | --- | --- |
|  | Thyroid nodules | GP | Thyroid nodules | GP | HR (95% C.I.) | |
| Anxiety disorder | 31576 | 34599 | 38.55 | 33.42 | 1.36 (1.34–1.38) |  |
| Mood disorder | 21472 | 26980 | 27.05 | 25.07 | 1.16 (1.14–1.18) |  |
| Psychotic disorder | 1455 | 2221 | 2.32 | 2.24 | 0.99 (0.93–1.06) |  |

GP: general population
